# Postoperative pancreatic fistula affects recurrence-free survival of pancreatic cancer patients

**DOI:** 10.1371/journal.pone.0252727

**Published:** 2021-06-04

**Authors:** Sameer A. Dhayat, Ahmad N. J. Tamim, Marius Jacob, Georg Ebeling, Laura Kerschke, Iyad Kabar, Norbert Senninger

**Affiliations:** 1 Department of General and Visceral Surgery, University Hospital Muenster, Muenster, Germany; 2 Institute of Biostatistics and Clinical Research, University of Muenster, Muenster, Germany; 3 Department of Internal Medicine B, Gastroenterology and Hepatology, University Hospital Muenster, Muenster, Germany; Ohio State University Wexner Medical Center Department of Surgery, UNITED STATES

## Abstract

**Purpose:**

Postoperative pancreatic fistula (POPF) with reported incidence rates up to 45% contributes substantially to overall morbidity. In this study, we conducted a retrospective evaluation of POPF along with its potential perioperative clinical risk factors and its effect on tumor recurrence.

**Methods:**

Clinical data on patients who had received pancreatoduodenectomy (PD), distal pancreatectomy (DP), or duodenum-preserving pancreatic head resection (DPPHR) were prospectively collected between 2007 and 2016. A Picrosirius red staining score was developed to enable morphological classification of the resection margin of the pancreatic stump. The primary end point was the development of major complications. The secondary end points were overall and recurrence-free survival.

**Results:**

340 patients underwent pancreatic resection including 222 (65.3%) PD, 87 (25.6%) DP, and 31 (9.1%) DPPHR. Postoperative major complications were observed in 74 patients (21.8%). In multivariable logistic regression analysis, POPF correlated with body mass index (BMI) (p = 0.025), prolonged stay in hospital (p<0.001), high Picrosirius red staining score (p = 0.049), and elevated postoperative levels of amylase or lipase in drain fluid (p≤0.001). Multivariable Cox regression analysis identified UICC stage (p<0.001), tumor differentiation (p<0.001), depth of invasion (p = 0.001), nodal invasion (p = 0.001), and the incidence of POPF grades B and C (p = 0.006) as independent prognostic markers of recurrence-free survival.

**Conclusion:**

Besides the known clinicopathological risk factors BMI and amylase in the drain fluid, the incidence of POPF correlates with high Picrosirius red staining score in the resection margins of the pancreatic stumps of curatively resected pancreatic ductal adenocarcinoma (PDAC). Furthermore, clinically relevant POPF seems to be a prognostic factor for tumor recurrence in PDAC.

## Introduction

Pancreatoduodenectomy (PD) is the preferred therapeutic method for treating benign and malignant diseases of the pancreatic head and its periampullary region. PD remains a complex and highly invasive visceral resection with mortality rates of about 5% and morbidity rates of up to 60% in experienced high-volume centres [1–3]. In line with the demographic changes resulting in an increasing number of multimorbid elderly patients in Europe and the USA, preoperative risk factor assessment and patient stratification have become increasingly popular. Postoperative pancreatic fistula (POPF) with reported incidence rates between 3% and 45% substantially contributes to overall morbidity with increased hospital stay, costs and reintervention rates and in case of abscess formation, sepsis, and hemorrhage to mortality as well [4,5]. Moreover, clinically relevant POPF with prolonged hospital stay may delay adjuvant treatment and affect oncologic outcomes in malignant pancreatic diseases [6].

The crucial importance of standardized reporting of procedures and their complications with uniform definition and classification of POPF and widely supported recommendations for its diagnosis and treatment led to a first consensus statement of the International Study Group of Pancreatic Surgery (ISGPS) in 2005 [7]. In 2017, clinically relevant POPF was redefined as drainage fluid of any measurable volume with amylase level more than three times that of physiological serum amylase activity, associated with a clinically relevant condition related directly to the POPF, which can originate from pancreaticoenteric or pancreaticogastric anastomosis after head resection or drainage procedures and of pancreatic remnant after distal pancreatectomy or enucleation [8].

The efficacy of anastomotic techniques like pancreaticojejunostomy versus pancreaticogastrostomy, invagination versus duct-to-mucosa, internal versus external pancreatic duct stenting, fibrin glue versus other topical haemostatic occlusive agents to seal the pancreatic anastomosis, and the use of various somatostatin analogues to decrease pancreatic enzyme secretion were investigated in various trials [9–16]. So far, there is no consensus on the optimal management of POPF and no standardized intra- and perioperative treatment [17]. Therefore, the attention has been focused on the assessment of POPF risk factors. Many studies addressed the role of single factors or comprehensive risk scores on multiple risk factors, such as age, gender, body mass index (BMI), pathologic diagnosis, operative time, blood loss, diameter of the main pancreatic duct, texture of pancreatic parenchyma, American Society of Anaesthesiologists (ASA) score, heart rate, systolic blood pressure, haemoglobin, and albumin levels [18]. POPF risk stratification customized for individual patients may help in increasing the number of patients eligible for pancreatic surgery by preoperative nutritional support, optimizing cardiovascular medication, starting exercise therapy, and postoperative intensive monitoring or even to select high-risk patients who might be excluded from surgical resection. However, current guidelines for pancreatic surgery do not recommend any of the proposed clinical risk prediction scores for morbidity and mortality such as ASA and POSSUM (Physiological and Operative Severity Score for the enUmeration of Mortality and Morbidity) nor POPF risk scores such as PREPARE (Preoperative Pancreatic Resection) score by Uzunoglu et al., FRS (Fistula Risk Score) by Callery et al., and a-FRS (Alternative Fistula Risk Score for Pancreatoduodenectomy) by Mungroop et al. [19–23].

In this study, we conducted a retrospective evaluation of POPF along with its potential perioperative clinical and histopathological risk factors and its impact on tumor recurrence.

## Patients and methods

### Study design

This retrospective monocentric study included patients who underwent pancreatic resection between January 2007 and December 2016 at the Department of General and Visceral Surgery of the University Hospital Muenster and were followed up for at least 36 months. All operative procedures were performed by surgeons experienced in the field of pancreatic surgery who conducted at least 30 pancreatic resections per year. Perioperative clinical data, histopathological information and follow-up data on all patients were prospectively collected in an electronic database. Ethical approval for postoperative tissue collection, analysis, and retrospective analysis of patient-related clinical data were obtained by the Ethics committee of the University Muenster (Az: 1IXHai v. 19.9.2001 and Az: 1IXHai v.11.08.2011) and all patients provided informed written consent. All patients underwent PD, left pancreatic resection, or duodenum-preserving pancreatic head resection. Patients that received emergency pancreatic resection, primary total pancreatectomy, immunosuppression, neoadjuvant chemotherapy, neoadjuvant radiotherapy, and/or missing follow-up data were excluded from this study.

The primary end point of the study was the development of major complications, in particular pancreatic fistula. The secondary end points were overall survival and recurrence free survival.

### Selection of perioperative variables

Perioperative variables included patient characteristics, medical history, physiological parameters, laboratory tests, and variables related to the performed surgery. Only variables that could be assessed objectively were accepted for inclusion. The cut off values based on physiological relevance or on established scoring systems.

Preoperative variables included age, gender, BMI, ASA status, heart rate, systolic blood pressure, biliary drainage, New York Heart Association (NYHA) Functional Classification, cardiac or pulmonary comorbidity, diabetes mellitus, smoking, alcohol abuse, weight loss over 10% in the last 6 months preceding the operation, acute or chronic pancreatitis, septic cholangitis, ascites, diagnosis, preoperative stay and other relevant comorbidities assessed by the Charlson comorbidity index [24]. Preoperative variables included standardized analyses of different blood parameters such as hemoglobin, leukocytes, electrolytes, creatinine, lipase, amylase, bilirubin, gamma-glutamyltransferase, transaminases, alkaline phosphatase, C-reactive protein (CRP), Cancer Antigen 19–9 (CA-19-9), and Carcinoembryonic antigen (CEA). Intraoperative variables included operation and anesthesia time, the set-up of an epidural catheter for continuous analgesia, heart rate, blood pressure, hemoglobin, blood transfusion, the surgical procedure, usage of internal or external drainages, kind of anastomosis, consistency of the pancreatic tissue, and the diameter size of the main duct at the pancreatic resection margin. Corresponding morphological features regarding the degree of fibrosis, inflammatory and fatty infiltration of the pancreatic resection margin of haematoxylin and eosin (H&E) stained specimens were also considered. Postoperative blood parameters included CRP, lipase, and amylase. In addition, lipase and amylase activities were assessed in the drainage fluid from postoperative day (POD) 3. Postoperative incidence and treatment of complications, such as pneumonia, heart attack, pulmonary embolism, thrombosis, urinary tract infection, wound infection, delayed gastric emptying, POPF, bile and chyle leakage, abdominal haemorrhage, and abscess were documented. Further data were obtained concerning the length of postoperative hospital stay particularly in the intensive care unit, reoperation, hospital mortality, the histopathological diagnosis, adjuvant chemotherapy regimens, tumor relapse, and tumor related deaths. In total, 72 variables were evaluated in this study and are presented in **Table 1**.

**Table 1:** Clinicopathological characteristics for postoperative complications.

	Total	No Complication	Complication		*p*-value
	n = 340	n = 117 (34.4%)	non severe (<IIIb) n = 149 (43.8%)	severe (≥IIIb) n = 74 (21.8%)	
**Age (years)**					
Median [range]	64 [15–88]	59 [15–86]	65 [15–88]	69.5 [20–82]	**0.002**
<60	144	64	57	23	
≥60	196	53	92	51	
**Gender**					
female	157	52	73	32	0.648
male	183	65	76	42	
**Body mass index (kg/m**^**2**^**)**					
Median [range]	24.6 [13.1–51.6]	24.4 [16.4–35.7]	24 [16.6–51.6]	25.9 [13.1–39.8]	**0.015**
18–24	165	60	80	25	
≥ 25	175	57	69	49	
**Smoking**					
No	268	88	118	62	0.385
Yes	72	29	31	12	
**Alcohol**					
No	310	100	140	70	**0.035**
Yes	30	17	9	4	
**Weight loss**					
No	311	103	138	70	0.377
Yes	29	14	11	4	
**ASA**					
2-Jan	247	87	113	47	0.141
4-Mar	93	30	36	27	
**NYHA**					
2-Jan	317	111	140	66	0.395
4-Mar	23	6	9	8	
**Cardiac comorbidity**					
No	161	69	67	25	**0.002**
Yes	179	48	82	49	
**Pulmonary comorbidity**					
No	305	112	133	60	**0.005**
Yes	35	5	16	14	
**Pre-op diabetes mellitus**					
No	252	88	112	52	0.706
Yes	88	29	37	22	
**Pre-op acute pancreatitis**					
No	325	110	146	69	0.128
Yes	15	7	3	5	
**Pre-op chronic pancreatitis**					
No	247	76	106	65	**0.001**
Yes	93	41	43	9	
**Pre-op septic cholangitis**					
No	326	115	140	71	0.289
Yes	14	2	9	3	
**Pre-op ascites**					
No	337	117	148	72	0.161
Yes	3	0	1	2	
**Charlson comorbidity index**					
0	27	11	14	2	0.016*
1	21	11	6	4	
2	146	56	68	22	
3	82	24	35	23	
4	35	9	14	12	
5	12	3	3	6	
≥6	17	3	9	5	
**Pre-op S-CA.19-9 (U/ml)**					
Median [range]		23 [0–15250]	27.5 [0.6–91630.3]	23 [0.6–25214]	0.92
< 30	187	66	80	41	
≥ 30	153	51	69	33	
**Pre-op S-CEA (ng/ml)**					
Median [range]		2.2 [1.4–83.6]	1.7 [0.2–284.4]	1.3 [0.1–3136.1]	0.45
<5	309	106	133	70	
≥5	31	11	16	4	
**Pre-op S-Creatinine (mg/dl)**					
Median [range]		0.8 [0.1–9]	0.8 [0.1–8]	0.9 [0.2–2]	0.764
≤1.25	300	105	131	64	
>1.25	40	12	18	10	
**Pre-op S-Bilirubin (mg/dl)**					
Median [range]		0.6 [0.2–7.8]	0.6 [0.1–24.3]	0.8 [0.2–15.6]	0.116
<1.1	253	90	115	48	
≥1.1	87	27	34	26	
**Pre-op S-Gamma-GT (U/l)**					
Median [range]		88 [9–1561]	74 [7–3530]	101.5 [8–1882]	0.4
<28	89	31	43	15	
≥28	251	86	106	59	
**Pre-op S-Alkaline phosphatase (U/l)**					
Median [range]		109 [33–1219]	101 [34–1731]	97.5 [16–1139]	0.243
<104	178	54	84	40	
≥104	162	63	65	34	
**Pre-op S-Amylase (U/l)**					
Median [range]		53.5 [13–267]	60 [8–615]	62.5 [17–585]	0.627
<40	217	71	99	47	
≥40	123	46	50	27	
**Pre-op S-Lipase (U/l)**					
Median [range]		50.5 [3–555]	43 [10–7000]	49 [7–468]	0.388
<60	223	71	102	50	
≥60	117	46	47	24	
**Pre-op S-CRP (mg/dl)**					
Median [range]		0.5 [0.5–30.8]	0.5 [0.1–32]	1 [0.4–22.8]	**0.037**
<8	314	107	143	64	
≥8	26	10	6	10	
**Pre-op P-Leukocytes (1/ μl)**					
Median [range]		6600 [1000–12500]	7000 [890–24060]	6980 [3250–23610]	0.612
<10,000	300	106	130	64	
≥10,000	40	11	19	10	
**Pre-op P-Hemoglobin (mg/dl)**					
Median [range]		11.9 [7.5–16.7]	11.4 [5.7–16.1]	10.9 [6.7–15.3]	**0.004**
<11.5	170	47	75	48	
11.5–17	170	70	74	26	
**Pre-op heart rate (1/min)**					
Median [range]		75 [40–100]	75 [50–110]	75 [55–120]	**0.025**
60–100	304	97	138	69	
<60 or >100	36	20	11	5	
**Pre-op systolic blood pressure (mmHg)**					
110–140	234	79	106	49	0.716
<110 or >140	106	38	43	25	
**Pre-op hospital stay (d)**					
Median [range]		1 [1–43]	1 [1–25]	1 [1–34]	0.145
<5	290	105	126	59	
≥5	50	12	23	15	
**Anaesthesia time (h)**					
Median [range]	7.75 [2.75–13]	8 [3.25–12.5]	7.3 [2.75–13]	8.125 [3.5–13]	**0.006**
<7	112	34	62	16	
≥7	228	83	87	58	
**Operation time (h)**					
Median [range]	6 [1.5–12]	6 [1.5–10.3]	5.75 [2–12]	6.275 [2.5–11.5]	0.104
<3.5	105	36	53	16	
≥3.5	235	81	96	58	
**Epidural anaesthesia**					
No	63	20	26	17	0.534
Yes	277	97	123	57	
**Intra-op blood transfusion**					
No	240	93	107	40	**0.001**
Yes	100	24	42	34	
**Surgical procedure**					
Traverso-Longmire/Whipple	222	72	91	59	**<0.001**
Distal pancreatectomy	87	24	50	13	
Drainage operation (Beger, Frey, Partington-Rochelle)	31	21	8	2	
**Pancreatojejunostomy**					
No	87	28	47	12	**0.042**
Yes	253	89	102	62	
**Internal/external drainage**					
No	256	98	109	49	**0.015**
Yes	84	19	40	25	
**Spleen resection**					
No	254	90	109	55	0.794
Yes	86	27	40	19	
**Pancreatic tissue**					
Soft	181	55	77	49	**0.03**
Hard	159	62	72	25	
**Main pancreatic duct (mm)**					
≤2	178	60	74	44	0.189
>2	162	57	75	30	
**Histological diagnosis**					
**malignant**					
Pancreatic Adenocarcinoma	126	46	60	20	0.209*
Papillary carcinoma	36	11	10	15	
NET	36	10	18	8	
Distal bile duct carcinoma	19	2	8	9	
Duodenal carcinoma	3	0	1	2	
Othertumors/metastasis	5	2	3	0	
**benign**					
Chronic pancreatitis	68	33	29	6	
IPMN	12	1	7	4	
Pancreatic cystadenoma	28	10	10	8	
Adenoma of the papilla Vateri	7	2	3	2	
**Tumor size (cm)**					
≤3	163	53	69	41	0.619
>3	62	18	31	13	
**UICC**					
I-II	95	31	41	23	0.904
III-IV	113	36	52	25	
**T**					
T1-T2	65	20	25	20	0.203
T3-T4	143	47	68	28	
**N**					
N0	100	33	43	24	
N1-2	108	34	50	24	
**M**					
M0	200	65	89	46	1
M1	8	2	4	2	
**L**					
L0	133	41	60	32	0.835
L1	75	26	33	16	
**V**					
V0	174	59	73	42	0.281
V1	34	8	20	6	
**G**					
G1	20	8	6	6	0.558
G2	116	35	57	24	
G3	72	24	30	18	
**R**					
R0	175	62	75	39	0.074
R1	33	5	18	9	
**Sirius red global score**					
A	85	40	35	10	<0.001*
B	81	18	40	23	
C	12	1	3	8	
**Stay in ICU/IMC (d)**					
Median [range]	6 [0–150]	5 [0–14]	6 [1–48]	14.5 [1–150]	**<0.001**
<6	163	77	74	12	
≥6	177	40	75	62	
**Total stay in hospital (d)**					
Median [range]	20 [2–377]	14 [8–43]	21 [9–67]	34 [2–377]	**<0.001**
<21	186	96	72	18	
≥21	154	21	77	56	
**Post-op S-Amylase (U/l)**					
Median [range]		62 [10–903]	59 [3–825]	78 [7–1035]	**0.025**
<40	196	77	85	34	
≥40	144	40	64	40	
**Amylase in drain fluid (U/l)**					
Median [range]		30 [6–815]	248 [2–7693]	827 [7–52277]	**<0.001**
<120	220	117	80	24	
≥120	120	0	69	50	
**Post-op S-Lipase (U/l)**					
Median [range]		39 [0–1088]	44.5 [1–1075]	68 [1–2648]	**0.017**
<60	204	72	98	34	
≥60	136	45	51	40	
**Lipase in drain fluid (U/l)**					
Median [range]		17.5 [4–1059]	236.5 [2–18940]	1935 [1–80561]	**<0.001**
<180	220	117	78	25	
≥180	120	0	71	49	
**Post-op S-CRP (mg/dl)**					
Median [range]	8.1 [0.5–45]	7.6 [0.5–32.9]	8 [0.5–45]	11 [0.5–39.8]	0.139
<8	166	63	74	29	
≥8	174	54	75	45	
**Post-op chemotherapy**					
No	112	26	46	40	**<0.001**
Yes	113	45	54	14	
**Tumor recurrence**					
No	111	34	48	29	0.782
Yes	114	37	52	25	
**Tumor related death**					
No	117	37	50	30	0.795
Yes	108	34	50	24	

Bold values indicate significance (p ≤ 0.05, Fisher’s exact test, Chi-Quadrat-test).

* ASA, American Society of Anesthesiologists; CA-19-9, Cancer Antigen 19–9; CEA, Carcinoembryonic antigen; CRP, C-reactive protein; d, day; Gamma-GT, Gamma-glutamyl transferase; ICU, intensive care unit; IMC, intermediate care unit; IPMN, intraductal papillary mucinous neoplasm; n, number of patients; NET, Neuroendocrine tumor; NYHA, New York Heart Association; op, operative; P, Plasma; S, Serum; UICC, Union for international cancer control.

### Definitions of postoperative complications

Major complications were defined according to the Clavien-Dindo classification as grades IIIb to V, validated for pancreatic resections [25,26]. Grade III includes complications requiring surgical, endoscopic or radiological intervention, followed by grade IV life-threatening complications requiring intensive care unit (ICU) management, and grade V as the complication-related death of the patient. POPF was defined according to the reclassification of the ISGPS in 2017 [8]. Depending on the clinical course three grades of severity were defined: 1. Biochemical leak (BL), former POPF grade A, with increased amylase activity in the fluid of a drain from POD 3 but without clinical implications. 2. POPF grade B that requires a change in the expected postoperative management with prolonged drainage, therapeutic agents and less-invasive treatment including percutaneous, endoscopic, or angiographic interventional procedures. 3. A shift to POPF grade C is characterized by organ failure, clinical instability, and high mortality, requiring reoperation and stay in an ICU.

Postoperative bile leakage was defined according to the definitions of the International Study Group of Liver Surgery (ISGLS) as discharge of fluid of an intra-abdominal drain with a bilirubin concentration at last three times greater than the serum bilirubin persisting on POD 3 [27]. Chyle leak after pancreatic resection was defined as the output of milky-colored fluid of a drain from POD 3 with a triglyceride content ≥110mg/dl [28]. In accordance with the definitions of the ISGPS, post-pancreatectomy hemorrhage was defined according to the time of onset, location, and clinical severity [29]. Delayed gastric emptying was diagnosed if the patient was unable to tolerate a solid diet on POD 7 or if reinsertion of the nasogastric tube was required between POD 4 and 7 according to the definitions of the ISGPS [30]. Mortality was defined as death occurring during the hospital stay.

### Histochemical staining by picrosirius red

**Fig 1:**
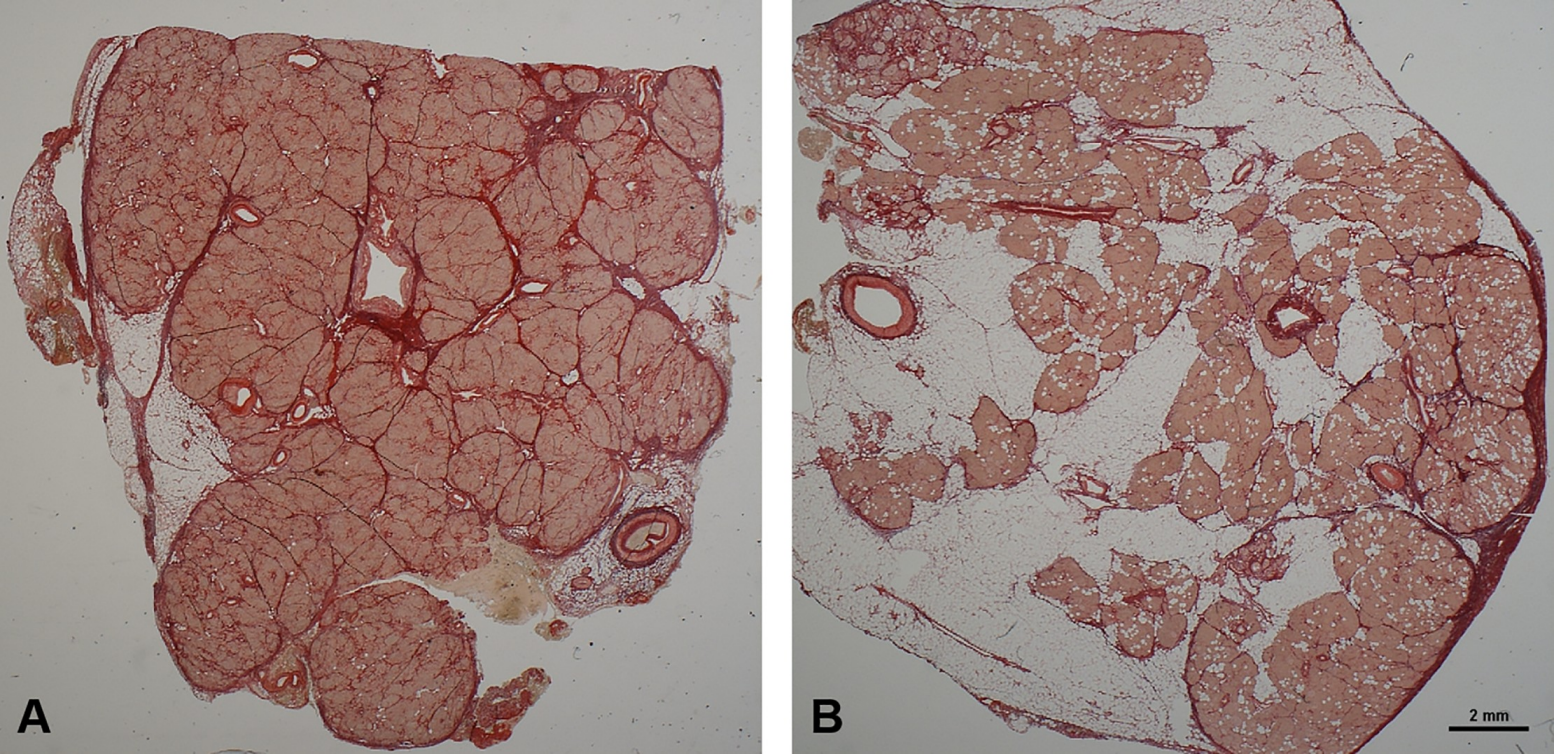
Histochemical staining by picrosirius red F3B. A: Resection margin of the pancreatic stump with focal perilobular and periacinar fibrosis, marginal steatosis, and absent inflammation. B: Resection margin of the pancreatic stump with generalized steatosis and marginal fibrosis.

The resection margins of pancreatic stumps of all cases treated by pancreatic head resection were submitted for pathological diagnosis by classic H&E staining. In addition, 6μm thick formalin-fixed paraffin embedded (FFPE) tissue sections of the resection margins were deparaffinized through xylene and a graded alcohol series. Tissue samples were stained for 30 min in Picrosirius red solution consisting of 0.1% solution of Sirius Red F3B (Sigma-Aldrich, St Louis, MO, USA) in saturated aqueous picric acid [31]. The stained sections were then washed for 2 min in 0.01 N HCI, dehydrated, cleared and mounted in synthetic resin. Negative controls without Sirius Red and positive controls (liver fibrosis and cirrhosis) were included in all experiments using the same experimental conditions. Picrosirius red planar staining was evaluated separately by two independent investigators in a blinded manner using light microscopy (Eclipse E1000M and NIS-Elements D3.1 imaging software, Nikon, Tokyo, Japan) with a modified score range from 1 to 3 according to Ridolfi et al. and Gaujoux et al. [32,33] Three grades of fibrosis were defined: 1. Normal pancreatic parenchyma, consisting in lobes separated by connective tissue organized in fine septa. 2. focal perilobular or periacinar fibrosis (**Fig 1A**), and 3. complete replacement of the parenchyma by fibrosis with rare residual areas of acinar glandular tissue. Similarly, the grade of inflammation by lymphocytes and of fatty infiltration by adipocytes was classified in three grades as absent, focal, and generalized (**Fig 1B**). Based on this, a global scoring system for morphological classification of the resection margin of the pancreatic stump was developed. Resulting from the sum of the values for each of the three morphological features of fibrosis, inflammatory and fatty infiltration, three groups A-C were defined: A: resection margin of the pancreatic stump with no or marginal alterations of inflammation and steatosis but generalized fibrosis (3–4), B: resection margin of the pancreatic stump with moderate alterations (5–6), and C: resection margin of the pancreatic stump with severe alterations of inflammation and steatosis but absent or marginal fibrosis (7–9).

### Statistical analysis

Statistical analyses were performed by SPSS Statistics Version 22 (IBM Corp. Armonk, NY) for Windows. Categorical parameters are reported as absolute and relative frequencies and continuous parameters as median [minimum-maximum]. All continuous variables were considered in a dichotomized form in further analyses. The association between clinicopathological parameters and the incidence of (no, non-severe, major) complications, and (no, BL, B, C) POPF was assessed by Fisher’s exact test. Uni- and multivariable logistic regression analyses were performed to identify independent risk factors for grade B/C POPF and major postoperative complications. All parameters with p≤0.05 were included in the multivariable analyses. Results are reported as odds ratios (OR), corresponding 95% confidence intervals (CI) and p-values.

Overall survival and recurrence free survival were measured from the date of surgery to the time of the last follow-up or cancer-related death or tumor relapse, respectively, considering patients who were still alive or without evidence of tumor relapse at the end of the study as censored. The Kaplan-Meier method was used to generate overall and recurrence-free survival curves of curatively treated PDAC patients with and without major postoperative complications and grade B/C POPF. Survival curves were compared by the log-rank test. A Cox proportional-hazards regression model was used to assess the impact of potential risk factors on overall and recurrence-free survival. All parameters with p≤0.05 were included in the multivariable models. A forward stepwise variable selection procedure based on the likelihood ratio test was applied. Results are reported as hazard ratios (HR), corresponding 95% CIs and p-values. All inferential statistics were intended to be exploratory. P≤0.05 was considered statistically noticeable.

## Results

### Clinicopathologic characteristics

A total of 340 patients underwent pancreatic resection during the study period including 222 (65.3%) PD according to either Traverso-Longmire (211; 95%) or Whipple-Kausch (11; 5%), 87 (25.6%) DP, and 31 (9.1%) DPPHR. The median age of the patients was 64 (15–88) years, their median BMI 24.6 (13.1–51.6) kg/m^2^, and 53.8% of patients were male. The distribution of various patient characteristics and physiological parameters by postoperative complication and POPF grade is shown in **Tables 1** and **2.** Postoperative complications were associated with age ≥60 years (p = 0.002), high BMI (p = 0.015), alcohol abuse (p = 0.035), cardiac and pulmonary comorbidity (p = 0.002; p = 0.005), absence of preoperative chronic pancreatitis (p = 0.001), high Charlson comorbidity index (p = 0.016), preoperative elevated serum level of CRP (p = 0.037), anemia (p = 0.004), preoperative heart rate up to 100/min (p = 0.025), prolonged anaesthesia time (p = 0.006), intraoperative blood transfusion (p = 0.001), pancreatojejunostomy procedure (p = 0.004), use of internal or external pancreatic duct drainage (p = 0.015), soft pancreatic tissue (p = 0.03), high Picrosirius red histochemical staining score (p<0.001), prolonged stay in ICU and hospital (each p<0.001), elevated postoperative serum levels of amylase (p = 0.025) and lipase (p = 0.017), as well as high concentrations of amylase and lipase (each p<0.001) in the drain fluid. POPF was associated with high BMI and ASA score (each p = 0.001), cardiac comorbidity (p = 0.011), absence of preoperative chronic pancreatitis (p<0.001), high preoperative bilirubin serum level (p = 0.023), preoperative heart rate up to 100/min (p = 0.01), pancreatojejunostomy procedure (p = 0.004), use of internal or external pancreatic duct drainage (p<0.001), soft pancreatic tissue (p<0.001), high Picrosirius red histochemical staining score (p<0.001), prolonged stay in ICU and hospital (each p<0.001), elevated postoperative serum levels of lipase (p = 0.017), as well as high concentrations of amylase and lipase (each p<0.001) in the drain fluid.

**Table 2:** Clinicopathological characteristics for postoperative pancreatic fistula.

	Total n = 340	w/o POPF n = 220 (64.7%)	Biochemical leak n = 59 (17.4%)	POPF B n = 30 (8.8%)	POPF C n = 31 (9.1%)	*p*-value
**Age (years)**						
Median [range]	64 (15–88)	64 (15–86)	63 (15–88)	62.5 (21–81)	70 (46–82)	0.395
<60	144	97	24	13	10	
≥60	196	123	35	17	21	
**Gender**						
Female	157	96	32	16	13	0.778
Male	183	124	27	14	18	
**Body mass index (kg/m**^**2**^**)**						
Median [range]	24.6 (13.1–51.6)	24.3 (16.4–41)	24.1 (13.1–51.6)	27.5 (19.2–35.6)	25.9 (18.6–37.6)	**0.001**
18–24	165	117	30	7	11	
≥25	175	103	29	23	20	
**Smoking**						
No	268	171	47	25	25	0.864
Yes	72	49	12	5	6	
**Alcohol**						
No	310	195	57	28	30	0.322
Yes	30	25	2	2	1	
**Weight loss**						
No	312	197	57	29	29	0.442
Yes	28	23	2	1	2	
**ASA**						
2-Jan	247	155	54	20	18	**0.001**
4-Mar	93	65	5	10	13	
**NYHA**						
2-Jan	315	206	55	27	27	0.561
4-Mar	25	14	4	3	4	
**Cardiac comorbidity**						
No	161	108	33	9	11	**0.011**
Yes	179	112	26	21	20	
**Pulmonary comorbidity**						
No	305	199	55	23	28	0.106
Yes	35	21	4	7	3	
**Pre-op diabetes mellitus**						
No	252	162	47	21	22	0.340
Yes	88	58	12	9	9	
**Pre-op acute pancreatitis**						
No	325	208	57	29	31	0.323
Yes	15	12	2	1	0	
**Pre-op chronic pancreatitis**						
No	247	147	45	27	28	**<0.001**
Yes	93	73	14	3	3	
**Pre-op septic cholangitis**						
No	327	209	59	29	30	1.000
Yes	13	11	0	1	1	
**Pre-op ascites**						
No	337	220	57	30	30	0.454
Yes	3	0	2	0	1	
**Charlson comorbidity index**						
0	27	18	8	1	0	0.051*
1	21	15	2	2	2	
2	146	96	30	12	8	
3	82	54	9	7	12	
4	35	19	6	6	4	
5	12	7	0	0	5	
≥	17	11	4	2	0	
**Pre-op S-CA.19-9 (U/ml)**						
Median [range]		33.65 (0.6–38085)	14.3 (0.6–1962.3)	39.2 (3.8–1675.4)	21.65 (0.6–3136.1)	0.888
< 30	187	113	41	13	20	
≥ 30	153	107	18	17	11	
**Pre-op S-CEA (ng/ml)**						
Median [range]		2 (0.2–83.4)	1.35 (0.2–284.4)	1 (01.11.8)	1.3 (0.2–3135.9)	1.000
<5	309	196	57	27	29	
≥5	31	24	2	3	2	
**Pre-op S-Creatinine(mg/dl)**						
Median [range]		0.8 (0.4–2.7)	0.8 (0.2–1.8)	0.9 (0.5–3.1)	0.9 (1.5–2)	0.826
≤1.25	300	193	52	27	28	
>1.25	40	27	7	3	3	
**Pre-op S-Bilirubin (mg/dl)**						
Median [range]		0.6 (0.1–24.3)	0.6 (0.2–9.7)	0.7 (0.2–9)	1 (0.3–7.7)	**0.023**
<1.1	253	168	47	20	18	
≥1.1	87	52	12	10	13	
**Pre-op S-Gamma-GT (U/l)**						
Median [range]		75 (7–3523)	80.5 (8–2367)	78 (12–1882)	161 (16–1091)	0.111
<28	89	62	17	5	5	
≥28	251	158	42	25	26	
**Pre-op S-Alkaline phosphatase (U/l)**						
Median [range]		104 (33–1731)	103 (16–834)	94.5 (38–592)	142 (35–1139)	0.889
<104	178	113	33	17	15	
≥104	162	107	26	13	16	
**Pre-op S-Amylase (U/l)**						
Median [range]		54 (8–272)	53 (14–615)	91.5 (23–585)	58.5 (36–217)	0.110
<40	216	144	39	15	18	
≥40	124	76	20	15	13	
**Pre-op S-Lipase (U/l)**						
Median [range]		45 (3–982)	51.5 (17–700)	55 (18–847)	48 (12–468)	0.658
<60	223	144	41	19	19	
≥60	117	76	18	11	12	
**Pre-op S-CRP (mg/dl)**						
Median [range]		0.5 (0.29–32)	0.5 (0.1–11.7)	0.5 (0.23–22.8)	1.2 (0.4–6.5)	1.000
<8	314	199	58	28	29	
≥8	26	21	1	2	2	
**Pre-op P-Leukocytes (1/ μl)**						
Median [range]		7.02 (0.89–24.06)	6.81 (2.5–16.3)	7.02 (4.25–22.09)	6.35 (3.25–9.8)	0.193
<10,000	300	191	52	26	31	
≥10,000	40	29	7	4	0	
**Pre-op P-Hemoglobin (mg/dl)**						
Median [range]		11.5 (5.7–16.7)	11.5 (7.1–15.7)	11.6 (7.2–16.1)	11 (6.7–14.9)	0.483
<11.5	170	108	28	13	21	
11.5–17	170	112	31	17	10	
**Pre-op heart rate (1/min)**						
Median [range]		75 (40–120)	80 (55–115)	70 (60–105)	70 (60–100)	**0.010**
60–100	304	192	52	29	31	
<60 or >100	36	28	7	1	0	
**Pre-surgical systolic blood pressure (mmHg)**						
110–140	234	149	43	22	20	1.000
<110 or >140	106	71	16	8	11	
**Pre-op hospital stay (d)**						
Median [range]		1 (1–43)	1 (1–23)	1 (1–34)	1 (1–17)	0.843
<5	290	187	50	27	26	
≥5	50	33	9	3	5	
**Anaesthesia time (h)**						
Median [range]	7.75 (2.75–13)	8 (2.75–12.5)	6.5 (4–13)	7.75 (4–11.75)	8.25 (3.5–11.5)	0.766
<7	112	61	33	12	6	
≥7	228	159	26	18	25	
**Operation time (h)**						
Median [range]	6 (1.5–12)	6 (1.5–12)	5 (2.25–11.5)	5.8 (2.5–9)	6.5 (3–8.75)	0.366
<3.5	105	62	28	11	4	
≥3.5	235	158	31	19	27	
**Epidural anaesthesia**						
No	63	41	7	7	8	0.209
Yes	277	179	52	23	23	
**Intra-op blood transfusion**						
No	240	149	49	24	18	0.878
Yes	100	71	10	6	13	
**Surgical procedure**						
Traverso-Longmire/Whipple	222	147	29	18	28	0.184
Distal pancreatectomy	87	48	27	9	3	
Drainage operation (Beger, Frey, Partington-Rochelle)	31	25	3	3	0	
**Pancreatojejunostomy**						
No	87	54	24	7	2	**0.004**
Yes	253	166	35	23	29	
**Internal/external drainage**						
No	256	183	40	16	17	**<0.001**
Yes	84	37	19	14	14	
**Spleen resection**						
No	254	161	44	24	25	0.262
Yes	86	59	15	6	6	
**Pancreatic tissue**						
Soft	181	96	40	21	24	**<0.001**
Hard	159	124	19	9	7	
**Main pancreatic duct (mm)**						
≤2	178	103	37	19	19	0.091
>2	162	117	22	11	12	
**Histological diagnosis**						
**malignant**						
Pancreatic Adenocarcinoma	126	100	14	6	6	**0.018**
Papillary carcinoma	36	15	11	4	6	
NET	36	16	11	6	3	
Distal bile duct carcinoma	19	10	2	2	5	
Duodenal carcinoma	3	1	0	1	1	
Othertumors/metastasis	5	2	2	1	0	
**benign**						
Chronic pancreatitis	68	55	9	2	2	
IPMN	12	4	3	4	1	
Pancreatic cystadenom	28	14	7	2	5	
Adenoma of the papilla Vateri	7	3	0	2	2	
**Tumor size (cm)**						
≤3	163	100	26	18	19	**0.012**
>3	62	44	13	2	3	
**UICC**						
I-II	95	57	21	9	8	0.714
III-IV	113	82	13	7	11	
**T**						
T1-T2	65	35	15	6	9	0.113
T3-T4	143	104	19	10	10	
**N**						
N0	100	60	22	10	8	0.712
N1-2	108	79	12	6	11	
**M**						
M0	200	134	32	15	19	1
M1	8	5	2	1	0	
**L**						
L0	133	87	25	10	11	0.699
L1	75	52	9	6	8	
**V**						
V0	174	114	29	13	18	0.462
V1	34	25	5	3	1	
**G**						
G1	20	10	6	1	3	0.922*
G2	116	77	20	12	7	
G3	72	52	8	3	9	
**R**						
R0	175	111	31	15	18	0.080
R1	33	28	3	1	1	
**Sirius red global score**						
A	85	76	7	1	1	**<0.001**
B	81	36	21	11	13	
C	12	1	1	6	4	
**Stay in ICU/IMC (d)**						
Median [range]	6 (0–147)	5 (0–34)	6 (1–39)	8.5 (2–48)	25 (3–147)	**<0.001**
<6	163	130	25	7	1	
≥6	177	90	34	23	30	
**Total stay in hospital (d)**						
Median [range]	20 (2–377)	16 (2–67)	20 (11–58)	30 (9–74)	46 (13–377)	**<0.001**
<21	186	150	31	3	2	
≥21	154	70	28	27	29	
**Post-op S-Amylase (U/l)**						
Median [range]		57 (0–903)	93.5 (15–825)	56.5 (10–340)	114 (18–1035)	0.069
<40	196	142	24	18	12	
≥40	144	78	35	12	19	
**Amylase in drain fluid (U/l)**						
Median [range]		25 (2–118)	674 (142–4500)	977 (202–25700)	1102 (138–52284)	**<0.001**
<120	220	220	0	0	0	
≥120	120	0	59	30	31	
**Post-op S-Lipase (U/l)**						
Median [range]		31.5 (0–1088)	62 (1–1075)	51 (14–2648)	167.5 (4–1099)	**<0.001**
<60	204	151	30	16	7	
≥60	136	69	29	14	24	
**Lipase in drain fluid (U/l)**						
Median [range]		17 (1–198)	2111.5 (231–18940)	3299.5 (400–33240)	4523.5 (138–80561)	**<0.001**
<180	220	220	0	0	0	
≥180	120	0	59	30	31	
**Post-op S-CRP (mg/dl)**						
Median [range]		7.9 (05.-45)	7.7 (0.5–27.6)	8.4 (1.3–27.5)	12.1 (1.3–38.5)	0.262
<8	166	108	32	15	11	
≥8	174	112	27	15	20	
**Post-op chemotherapy**						
No	113	58	21	14	20	**<0.001**
Yes	112	86	18	6	2	
**Tumor recurrence**						
No	112	69	25	11	7	0.392
Yes	113	75	14	9	15	
**Tumor related death**						
No	118	72	26	13	7	0.498
Yes	107	72	13	7	15	
**Insufficiency of pancreatojejunostomy**						
No	220	220	0	0	0	**<0.001**
Yes	120	0	59	30	31	
**Hemorrhage**						
No	310	212	54	26	18	**<0.001**
Yes	30	8	5	4	13	
**Abscess**						
No	313	214	56	21	22	**<0.001**
Yes	27	6	3	9	9	
**Bile leak**						
No	324	218	58	30	18	**<0.001**
Yes	16	2	1	0	13	
**Insufficiency of duodenojejunostomy**						
No	321	216	58	30	17	**<0.001**
Yes	19	4	1	0	14	
**Lymphatic fistula**						
No	325	213	57	29	26	**0.035**
Yes	15	7	2	1	5	
**Pneumonia**						
No	287	208	52	19	8	**<0.001**
Yes	53	12	7	11	23	
**Urinary tract infection**						
No	306	204	54	26	22	**0.003**
Yes	34	16	5	4	9	
**Wound infection**						
No	270	194	52	18	6	**<0.001**
Yes	70	26	7	12	25	
**Delayed gastric emptying**						
No	235	175	42	16	2	**<0.001**
Yes	105	45	17	14	29	
**Thrombosis**						
No	329	216	58	27	28	**0.006**
Yes	11	4	1	3	3	
**Pulmonary embolism**						
No	327	216	56	28	27	**0.016**
Yes	13	4	3	2	4	
**Heart attack**						
No	336	218	59	29	30	0.149
Yes	4	2	0	1	1	
**Complication grade (Clavien-Dindo)**						
0	117	117	0	0	0	**<0.001**
I	48	21	27	0	0	
II	91	56	20	15	0	
IIIa	10	3	2	5	0	
IIIb	24	12	6	6	0	
Iva	7	1	3	1	2	
IVb	23	2	1	3	17	
V	20	8	0	0	12	

Bold values indicate significance (p ≤ 0.05, Fisher’s exact test Chi-Quadrat-test).

* ASA, American Society of Anesthesiologists; CA-19-9, Cancer Antigen 19–9; CEA, Carcinoembryonic antigen; CRP, C-reactive protein; d, day; Gamma-GT, Gamma-glutamyl transferase; ICU, intensive care unit; IMC, intermediate care unit; IPMN, intraductal papillary mucinous neoplasm; n, number of patients; NET, Neuroendocrine tumor; NYHA, New York Heart Association; op, operative; P, Plasma; S, Serum; UICC, Union for international cancer control.

Pancreatic anastomosis after PD was performed by duct-to-mucosa and end-to-side pancreaticojejunostomy in all patients. A surgical drain was placed in all patients. PD with pancreatic duct drainage was performed internally in 92.5% (n = 74) and externally in 7.5% (n = 6). The leading indication for surgery was malignancy (n = 225; 66.2%) including pancreatic ductal adenocarcinoma (PDAC) (n = 126; 56%), neuroendocrine carcinoma (n = 36; 16%), cancer of the ampulla of Vater (n = 36; 16%), distal bile duct cancer (n = 19; 8.4%), duodenal adenocarcinoma (n = 3; 1.3%), and other tumors (n = 5; 2.2%). A minority of the study cohort underwent surgery because of chronic pancreatitis (n = 68; 20%), IPMN (n = 12; 3.5%), or adenoma (n = 35; 10.3%). Median operative time was 390 min (range 210–690 min) for PD, 240 min (range 120–720 min) for DP, and 270 min (range 90–450 min) for DPPHR, respectively. A total of 100 patients (29.4%) received an intraoperative blood transfusion. 100μg of Octreotide was subcutaneously administered TDS for five days postoperatively in all patients. The mean length of hospital and intensive care unit stay was 20 and 6 days, respectively. The 30 days mortality rate was 5% (n = 17) in total, 6.3% (n = 14) in the PD, 3.4% (n = 3) in the DP, and 0% in the DPPHR group.

### Morbidity risk factors

The overall morbidity rate was 65.6% (n = 223) in total, with 67.6% (n = 150) in the PD, 72.4% (n = 63) in the DP, and 32.3% (n = 10) in the DPPHR group. Postoperative minor complications defined as grades I-IIIa according to Clavien-Dindo were identified in 149 cases (43.8%) with 41.0% (n = 91) in the PD, 57.5% (n = 50) in the DP, and 25.8% (n = 8) in the DPPHR group. Delayed gastric emptying was detected in 70.5% (n = 105), abdominal wound infection in 47.0% (n = 70), pneumonia in 35.6% (n = 53), urinary tract infection in 22.8% (n = 34), thrombosis in 7.4% (n = 11), pulmonary embolism in 8.7% (n = 13), and heart attack in 4 cases (2.7%). Postoperative major complications defined as grades IIIb-V according to Clavien-Dindo were observed in 74 patients (21.8%) with 26.6% (n = 59) in the PD, 14.9% (n = 13) in the DP, and 6.5% (n = 2) in the DPPHR group. Bile leakage was detected in 21.6% (n = 16), POPF in 82.4% (n = 61), insufficiency of the duodenojejunostomy in 25.7% (n = 19), chyle leak in 20.3% (n = 15), and abdominal bleeding in 40.5% (n = 30). A total of 13 patients (3.8%) underwent a second operation due to abdominal bleeding associated with POPF. Of all patients, 59 (17.4%) were affected by BL, 30 (8.8%) by POPF grade B, and 31 (9.1%) by POPF grade C. The overall incidence of clinically relevant POPF was 17.9% (n = 61).

In the univariable logistic regression analysis of main perioperative patient characteristics, age ≥60 years (p = 0.028), increased BMI (p = 0.005), ASA 3–4, (p = 0.048), Charlson comorbidity index ≥3 (p<0.001), cardiac and pulmonary comorbidity (p = 0.009; p = 0.007), absence of preoperative chronic pancreatitis (p = 0.002), preoperative elevated levels of bilirubin (p = 0.035) and CRP in blood serum (p = 0.036), plasma haemoglobin levels <11.5 or >17 g/dl (p = 0.004), intraoperative blood transfusion (p = 0.001), prolonged anaesthesia time ≥7 hours (p = 0.021), no use of internal or external pancreatic duct drainage (p = 0.042), PD as surgical procedure (p = 0.039), soft pancreatic tissue (p = 0.012), high Picrosirius red histochemical staining score (p = 0.001), prolonged stay at hospital and at ICU (p<0.001), and elevated levels of postoperative amylase and lipase in drain fluid and blood serum (each p<0.03) were associated with an increased probability of major postoperative complications **(Table 3)**. Multivariable logistic regression analysis indicated a correlation between major complications after pancreatic surgery and pulmonary comorbidity (p = 0.005), high BMI (p = 0.023), preoperative CRP of ≥8 mg/l (p = 0.04), plasma haemoglobin levels <11.5 or >17 g/dl (p = 0.041), prolonged anesthesia time (p = 0.018), stay at ICU ≥7 days (p = 0.008), and elevated amylase and lipase activity in the drainage fluid (each p<0.001).

**Table 3:** Uni- and multivariable logistic regression analysis of risk factors for major (grade IIIb to V) postoperative complications.

Variable	Subset	Univariate analysis		Multivariate analysis	
		OR [95% CI]^1,2^	*p*	OR [95% CI]	*p*
**Age**	<60 vs ≥60 years	0.540 (0.312–0.935)	**0.028**	1.018 (0.478–2.168)	0.963
**Gender**	Female vs male	0.859 (0.511–1.444)	0.567		
**Body mass index**	<18 or >25 vs 18–25 kg/m^2^	2.178 (1.271–3.731)	**0.005**	2.112 (1.110–4.021)	**0.023**
**Smoking**	Yes vs No	1.505 (0.761–2.976)	0.240		
**Alcohol**	No vs Yes	0.527 (0.178–1.562)	0.248		
**Weight loss**	No vs Yes	0.576 (0.193–1.716)	0.322		
**ASA**	1–2 vs 3–4	0.574 (0.332–0.995)	**0.048**	0.769 (0.350–1.691)	0.513
**NYHA**	1–2 vs 3–4	0.493 (0.200–1.213)	0.123		
**Cardiac comorbidity**	No vs Yes	0.488 (0.285–0.836)	**0.009**	0.923 (0.422–1.926)	0.830
**Pulmonary comorbidity**	Yes vs No	2.722 (1.308–5.665)	**0.007**	3.568 (1.481–8.595)	**0.005**
**Pre-op diabetes mellitus**	No vs Yes	0.780 (0.441–1.380)	0.394		
**Pre-op acute pancreatitis**	No vs Yes	1.855 (0.614–5.606)	0.273		
**Pre-op chronic pancreatitis**	No vs Yes	3.333 (1.585–7.011)	**0.002**	1.250 (0.462–3.383)	0.660
**Pre-op septic cholangitis**	No vs Yes	1.077 (0.289–4.020)	0.912		
**Pre-op ascites**	Yes vs No	7.361 (0.658–82.332)	0.105		
**Charlson comorbidity index**	≥3 vs <3	1.365 (1.153–1.617)	**<0.001**	1.126 (0.873–1.452)	0.359
**Pre-op S-CEA**	<4.6 vs ≥4.6 ng/ml	1.977 (0.669–5.841)	0.218		
**Pre-op S-CA.19-9**	<37 vs ≥37 U/ml	1.021 (0.608–1.714)	0.937		
**Pre-op S-Creatinine**	<1.25 vs ≥1.25 mg/dl	0.814 (0.378–1.752)	0.598		
**Pre-op S-Bilirubin (mg/dl)**	<1,1 vs ≥1.1 mg/dl	0.549 (0.315–0.958)	**0.035**	1.183 (0.534–2.623)	0.678
**Pre-op S-Gamma-GT**	< 28 U/l vs ≥28 U/l	0.660 (0.352–1.235)	0.192		
**Pre-op S-Alkaline phosphatase**	<175 vs ≥175 U/l	1.091 (0.651–1.829)	0.740		
**Pre-op S-Amylase**	<40 vs ≥40 U/l	0.983 (0.576–1.679)	0.950		
**Pre-op S-Lipase**	<60 vs ≥60 U/l	1.120 (0.647–1.937)	0.685		
**Pre-op S-CRP**	<8 vs ≥8 mg/l	0.410 (0.177–0.945)	**0.036**	0.330 (0.115–0.951)	**0.040**
**Pre-op P-Leukocytes**	10.000 vs ≥10.000 /μl	0.814 (0.378–1.752)	0.598		
**Pre-op P-Hemoglobin**	11.5–17 vs <11.5 or >17 g/dl	0.459 (0.269–0.783)	**0.004**	1.960 (1.027–3.739)	**0.041**
**Pre-op systolic blood pressure**	110–140 vs <110 or >140 mmHg	0.858 (0.496–1.484)	0.584		
**Pre-op heart rate**	60–100 vs <60 or >100/min	1.820 (0.682–4.860)	0.232		
**Intra-op blood transfusion**	Yes vs No	2.576 (1.508–4.399)	**0.001**	0.783 (0.330–1.861)	0.580
**Pre-op hospital stay**	<5 vs ≥5d	0.596 (0.305–1.164)	0.129		
**Anesthesia time**	<7 vs ≥7 h	0.489 (0.266–0.897)	**0.021**	0.404 (0.191–0.855)	**0.018**
**Operating time**	<3.5 vs ≥3.5 h	0.549 (0.298–1.009)	0.053		
**Epidural anesthesia**	No vs Yes	0.701 (0.374–1.314)	0.268		
**Spleen resection**	No vs Yes	1.026 (0.568–1.852)	0.932		
**Internal/external drainage**	No vs Yes	0.559 (0.319–0.980)	**0.042**	1.537 (0.622–2.958)	0.443
**Operation procedure**	Left resection vs Whipple/TLM	0.485 (0.251–0.939)	**0.032**	2.708 (0.409–17.937)	0.302
	Drainage operation vs Whipple/TLM	0.191 (0.044–0.823)	**0.026**	1.360 (0.147–12.542)	0.786
**Pancreatojejunostomy**	Yes vs No	2.029 (1.035–3.978)	**0.039**	0.846 (0.210–3.418)	0.815
**Main pancreatic duct**	≤2 vs >2 mm	0.692 (0.410–1.167)	0.168		
**Pancreatic tissue**	Soft vs hard	1.990 (1.161–3.409)	**0.012**	1.311 (0.624–2.753)	0.474
**Histological diagnosis**	Malignant vs benign	1.500 (847–2.655)	0.164		
	PDAC vs Chronic pancreatitis	1.862 (710–4.881)	0.206		
**Tumor size**	≤3 vs >3mm	1.088 (0.588–2.011)	0.789		
**UICC**	3–4 vs 1–2	1.105 (0.580-2-107)	0.761		
**T**	T3-4 vs T1-2	1.825 (0.935–3.565)	0.078		
**N**	N1-2 vs N0	1.120 (0.587–2.136)	0.731		
**G**	G3-4 vs G1-2	1.003 (0.510–1.973)	0.993		
**R**	R1-2 vs R0	0.788 (0.338–1.835)	0.580		
**L**	L1 vs L0	1.203 (0.606–2.388)	0.597		
**V**	V1 vs V0	1.585 (0.615–4.088)	0.340		
**M**	M1 vs M0	0.860 (0.168–4.415)	0.857		
**Stay in ICU/IMC**	<7 vs ≥7d	0.147 (0.076–0.286)	**<0.001**	0.343 (0.157–0.752)	**0.008**
**Total stay in hospital**	<21 vs ≥21d	0.188 (0.104–0.377)	**<0.001**	0.488 (0.237–1.005)	0.051
**Post-op S-Amylase**	<40 vs ≥40 U/l	0.546 (0.325–0.917)	**0.022**	0.620 (0.315–1.222)	0.168
**Amylase in drain fluid**	<120 vs ≥120 U/l	0.171 (0.098–0.300)	**<0.001**	0.192 (0.095–0.386)	**<0.001**
**Post-op S-Lipase**	<60 vs ≥60 U/l	0.480 (0.285–0.808)	**0.006**	0.834 (0.390–1.783)	0.639
**Lipase in drain fluid**	<180 vs ≥180 U/l	0.186 (0.107–0.323)	**<0.001**	0.158 (0.075–0.335)	**<0.001**
**Post-op S-CRP**	<8 vs ≥8 mg/l	0.607 (0.359–1.026)	0.062		
**Sirius red global score**	A vs B/C	0.267 (0.121–0.587)	**0.001**	1.088 (0.381–3.106)	0.874

Bold values indicate significance (p ≤ 0.05). ASA, American Society of Anesthesiologists; CA-19-9, Cancer Antigen 19–9; CEA, Carcinoembryonicantigen; CI, confidence interval; CRP, C-reactive protein; d, day; Gamma-GT, Gamma-glutamyl transferase; OR, Odds ratio; ICU, intensive care unit; IMC, intermediate care unit; NYHA, New York Heart Association; op, operative; P, Plasma; S, Serum; UICC, Union for International Cancer Control.

In addition univariable logistic regression analysis showed a correlation between POPF (grade B/C) incidence and high BMI (p = 0.001), elevated ASA score (p = 0.028), Charlson comorbidity index ≥3 (p = 0.047), cardiac comorbidity (p = 0.009), absence of preoperative chronic pancreatitis (p = 0.001), elevated levels of preoperative bilirubin (p = 0.023), preoperative tachycardia (p = 0.034), no use of internal or external pancreatic duct drainage (p<0.001), soft pancreatic tissue (p<0.001), high histochemical staining score (p<0.001), prolonged stay in ICU and hospital (each p<0.001), and elevated postoperative levels of amylase and lipase in drain fluid (each p<0.001) **(Table 4)**. Moreover, BMI (p = 0.025), high Picrosirius red global score (p = 0.049), prolonged stay in hospital (p<0.001), and elevated postoperative levels of amylase and lipase in drain fluid (p≤0.001) were identified as potential independent risk factors of POPF in multivariable logistic regression analysis.

**Table 4:** Uni- and multivariable logistic regression analysis of risk factors for postoperative pancreatic fistula (B/C).

Variable	Subset	Univariate analysis		Multivariate analysis	
		OR [95% CI]^1,2^	*p*	OR [95% CI]	*p*
**Age**	<60 vs ≥60 years	0.765 (0.434–1.349)	0.355		
**Sex**	Female vs male	1.115 (0.649–1.934)	0.699		
**Body mass index**	<18 or >25 vs 18–25 kg/m^2^	2.743 (1.510–4.982)	**0.001**	2.892 (1.143–7.318)	**0.025**
**Smoking**	Yes vs No	1.147 (0.574–2.291)	0.698		
**Alcohol**	No vs Yes	0.473 (0.139–1.611)	0.231		
**Weight loss**	No vs Yes	0.515 (0.150–1.762)	0.290		
**ASA**	1–2 vs 3–4	0.523 (0.293–0.933)	**0.028**	0.212 (0.043–1.037)	0.055
**NYHA**	1–2 vs 3–4	0.480 (0.188–1.222)	0.124		
**Cardiac comorbidity**	No vs Yes	0.463 (0.259–0.828)	**0.009**	0.597 (0.223–1.603)	0.306
**Pulmonary comorbidity**	Yes vs No	1.946 (0.882–4.296)	0.099		
**Pre-op diabetes mellitus**	No vs Yes	0.747 (0.408–1.369)	0.345		
**Pre-op acute pancreatitis**	No vs Yes	0.309 (0.040–2.396)	0.261		
**Pre-op chronic pancreatitis**	No vs Yes	4.251 (1.765–10.242)	**0.001**	1.725 (0.373–7.976)	0.485
**Pre-op septic cholangitis**	No vs Yes	0.806 (0.174–3.732)	0.783		
**Pre-op ascites**	Yes vs No	2.262 (0.202–25.351)	0.508		
**Charlson comorbidity index**	≥3 vs <3	1.162 (1.002–1.348)	**0.047**	0.872 (0.567–1.343)	0.534
**Pre-op S-CEA**	<4.6 vs ≥4.6 ng/ml	1.176 (0.433–3.195)	0.750		
**Pre-op S-CA.19-9**	<37 vs ≥37 U/ml	0.992 (0.571–1.725)	0.977		
**Pre-op S-Creatinine**	<1.25 vs ≥1.25 mg/dl	1.058 (0.445–2.517)	0.898		
**Pre-op S-Bilirubin (mg/dl)**	<1,1 vs ≥1.1 mg/dl	0.507 (0.282–0.911)	**0.023**	1.112 (0.372–7.976)	0.849
**Pre-op S-Gamma-GT**	< 28 U/l vs ≥28 U/l	0.533 (0.274–1.116)	0.098		
**Pre-op S-Alkaline phosphatase**	<175 vs ≥175 U/l	1.044 (0.601–1.812)	0.879		
**Pre-op S-Amylase**	<40 vs ≥40 U/l	0.630 (0.361–1.102)	0.105		
**Pre-op S-Lipase**	<60 vs ≥60 U/l	0.866 (0.489-1-535)	0.623		
**Pre-op S-CRP**	<8 vs ≥8 mg/l	1.246 (0.414–3.754)	0.696		
**Pre-op P-Leukocytes**	10.000 vs ≥10.000 /μl	2.157 (0.738–6.301)	0.160		
**Pre-op P-Hemoglobin**	11.5–17 vs <11.5 or >17 g/dl	1.268 (0.730–2.203)	0.400		
**Pre-op systolic blood pressure**	110–140 vs <110 or >140 mmHg	1.031 (0.568–1.872)	0.920		
**Pre-op heart rate**	60–100 vs <60 or >100/min	0.114 (0.015–0.847)	**0.034**	0.112 (0.012–1.050)	0.055
**Intra-op blood transfusion**	Yes vs No	1.075 (0.591–1.956)	0.814		
**Pre-op hospital stay**	<5 vs ≥5d	1.201 (0.533–2.705)	0.658		
**Anesthesia time**	<7 vs ≥7 h	0.879 (0.485–1.593)	0.671		
**Operating time**	<3.5 vs ≥3.5 h	0.739 (0.396–1.376)	0.340		
**Epidural anesthesia**	No vs Yes	0.637 (0.329–1.234)	0.181		
**Spleen resection**	No vs Yes	0.678 (0.342–1.346)	0.267		
**Internal/external drainage**	No vs Yes	0.306 (0.172–0.547)	**<0.001**	0.668 (0.251–1.780)	0.419
**Operation procedure**	Left resection vs Whipple/TLM	0.627 (0.343–1.317)	0.247		
	Drainage operation vs Whipple/TLM	0.410 (0.119–1.408)	0.157		
**Pancreatojejunostomy**	Yes vs No	1.992 (0.964–4.117)	0.063		
**Main pancreatic duct**	≤2 vs >2 mm	0.590 (0.335–1.039)	0.068		
**Pancreatic tissue**	Soft vs hard	3.045 (1.646–5.636)	**<0.001**	1.400 (0.382–5.125)	0.611
**Histological diagnosis**	Malignant vs benign	1.058 (0.587–1.908)	0.850		
	PDAC vs Chronic pancreatitis	1.763 (0.522–5.630)	0.399		
**Tumor size**	≤3 vs >3mm	1.590 (0.783–3.229)	0.199		
**UICC**	3–4 vs 1–2	1.204 (0.582–2.494)	0.616		
**T**	T3-4 vs T1-2	1.845 (0.875–3.890)	0.107		
**N**	N1-2 vs N0	1.190 (0.575–2.462)	0.640		
**G**	G3-4 vs G1-2	1.009 (0.467–2.181)	0.981		
**R**	R1-2 vs R0	0.774 (0.362–1.652)	0.507		
**L**	L1 vs L0	3.707 (0.844–16.274)	0.083		
**V**	V1 vs V0	1.641 (0.538–5.006)	0.385		
**M**	M1 vs M0	1.444 (0.172–12.131)	0.735		
**Stay in ICU/IMC**	<7 vs ≥7d	0.137 (0.065–0.288)	**<0.001**	0.347 (0.113–1.063)	0.064
**Total stay in hospital**	<21 vs ≥21d	0.047 (0.018–0.121)	**<0.001**	0.123 (0.040–0.383)	**<0.001**
**Post-op S-Amylase**	<40 vs ≥40 U/l	0.633 (0.364-1-099)	0.104		
**Amylase in drain fluid**	<120 vs ≥120 U/l	0.005 (0.001–0.034)	**<0.001**	0.177 (0.034–0.107)	**0.001**
**Post-op S-Lipase**	<60 vs ≥60 U/l	0.344 (0.195–0.606)	**<0.001**	0.578 (0.199–1.682)	0.314
**Lipase in drain fluid**	<180 vs ≥180 U/l	0.004 (0.001–0.033)	**<0.001**	0.005 (0.001–0.044)	**<0.001**
**Post-op S-CRP**	<8 vs ≥8 mg/l	0.712 (0.408–1.242)	0.231		
**Sirius red global score**	A vs B/C	0.042 (0.010–0.181)	**<0.001**	0.138 (0.019–0.991)	**0.049**

Bold values indicate significance (p ≤ 0.05). ASA, American Society of Anesthesiologists; CA-19-9, Cancer Antigen 19–9; CEA, Carcinoembryonic antigen; CI, confidence interval; CRP, C-reactive protein; d, day; Gamma-GT, Gamma-glutamyl transferase; OR, Odds ratio; ICU, intensive care unit; IMC, intermediate care unit; NYHA, New York Heart Association; op, operative; P, Plasma; S, Serum; UICC, Union for international cancer control.

Further multivariable logistic regression analyses showed a correlation of clinically relevant POPF and the postoperative major and minor complications, such as bile leakage (p = 0.003), abdominal haemorrhage and abscess (each p<0.001), pneumonia (p<0.001), and wound infection (p = 0.003) **(Table 5)**.

**Table 5:** Uni- and multivariable logistic regression analysis of the association between postoperative pancreatic fistula (B/C) and other post-surgical complications.

Variable	Subset	Univariate analysis		Multivariate analysis	
		OR [95% CI]^1,2^	*p*	OR [95% CI]	*p*
**Hemorrhage**	No vs Yes	0.078 (0.037–0.164)	**<0.001**	0.136 (0.049–0.373)	**<0.001**
**Abscess**	No vs Yes	0.082 (0.035–0.194)	**<0.001**	0.087 (0.030–0.255)	**<0.001**
**Bile Leak**	No vs Yes	0.041 (0.011–0.150)	**<0.001**	0.92 (0.019–0.456)	**0.003**
**Insufficiency of duodenojejunostomy**	No vs Yes	0.063 (0.022–0.182)	**<0.001**	0.414 (0.0.53–3.214)	0.399
**Lymphatic fistula**	No vs Yes	0.312 (0.107–0.913)	**0.033**	2.261 (0.364–14.034)	0.381
**Pneumonia**	No vs Yes	0.060 (0.030–0.120)	**<0.001**	0.151 (0.061–0.372)	**<0.001**
**Urinary tract infection**	No vs Yes	0.308 (0.145–0.656)	**0.002**	0.574 (0.162–2.029)	0.389
**Wound infection**	No vs Yes	0.082 (0.044–0.154)	**<0.001**	0.272 (0.115–0.647)	**0.003**
**Delayed gastric emptying**	No vs Yes	0.155 (0.062–0.213)	**<0.001**	0.865 (0.312–2.393)	0.779
**Thrombosis**	No vs Yes	0.171 (0.050–0.580)	**0.005**	1.021 (0.141–7.386)	0.984
**Pulmonary embolism**	No vs Yes	0.241 (0.078–0.745)	**0.013**	1.024 (0.185–5.675)	0.979
**Heart attack**	No vs Yes	0.217 (0.030–1.574)	0.131	0.122 (0.006–2.345)	0.164
**Complication grade (Clavien-Dindo)**	<3b vs ≥3b	0.062 (0.032–0.188)	**<0.001**	0.461 (0.160–1.329)	0.152

Bold values indicate significance (p ≤ 0.05). ASA, American Society of Anesthesiologists; CA-19-9, Cancer Antigen 19–9; CEA,Carcinoembryonic antigen;CI, confidence interval; CRP, C-reactive protein;d, day; Gamma-GT, Gamma-glutamyl transferase; HR, hazard ratio;ICU, intensive care unit; IMC, intermediate care unit; NYHA, New York Heart Association; op, operative; P, Plasma; S, Serum;UICC, Union for international cancer control.

Among patients that developed a POPF grade B (n = 30; 8.8%), a total of 28 (93.3%) were treated by computed tomography guided percutaneous drainage for infected intra-abdominal fluid collections and 3 angiographic procedures with successful coil embolization of a ruptured gastroduodenal artery pseudoaneurysm were performed. Surgical reoperations were required in 31 patients with POPF grade C (9.1%). In detail, 12 cases (38.7%) with recreation of the pacreaticojejunostomy, 4 cases (12.9%) with recreation of the pancreaticojejunostomy as well as the biliodigestive anastomosis, 7 cases (22.6%) with recreation of the pancreaticojejunostomy as well as the biliodigestive anastomosis followed by relaparotomy with resection of the pancreatic stump, 3 cases (9.7%) with primary resection of the pancreatic stump, 4 cases (12.9%) with intraabdominal haematoma removal and resection of the bleeding pseudoaneurysm of the gastroduodenal artery stump, and 1 case (0.9%) with relaparotomy and removal of abdominal abscess formation were performed as postoperative complication management.

### Overall survival and tumor recurrence

**Fig 2:**
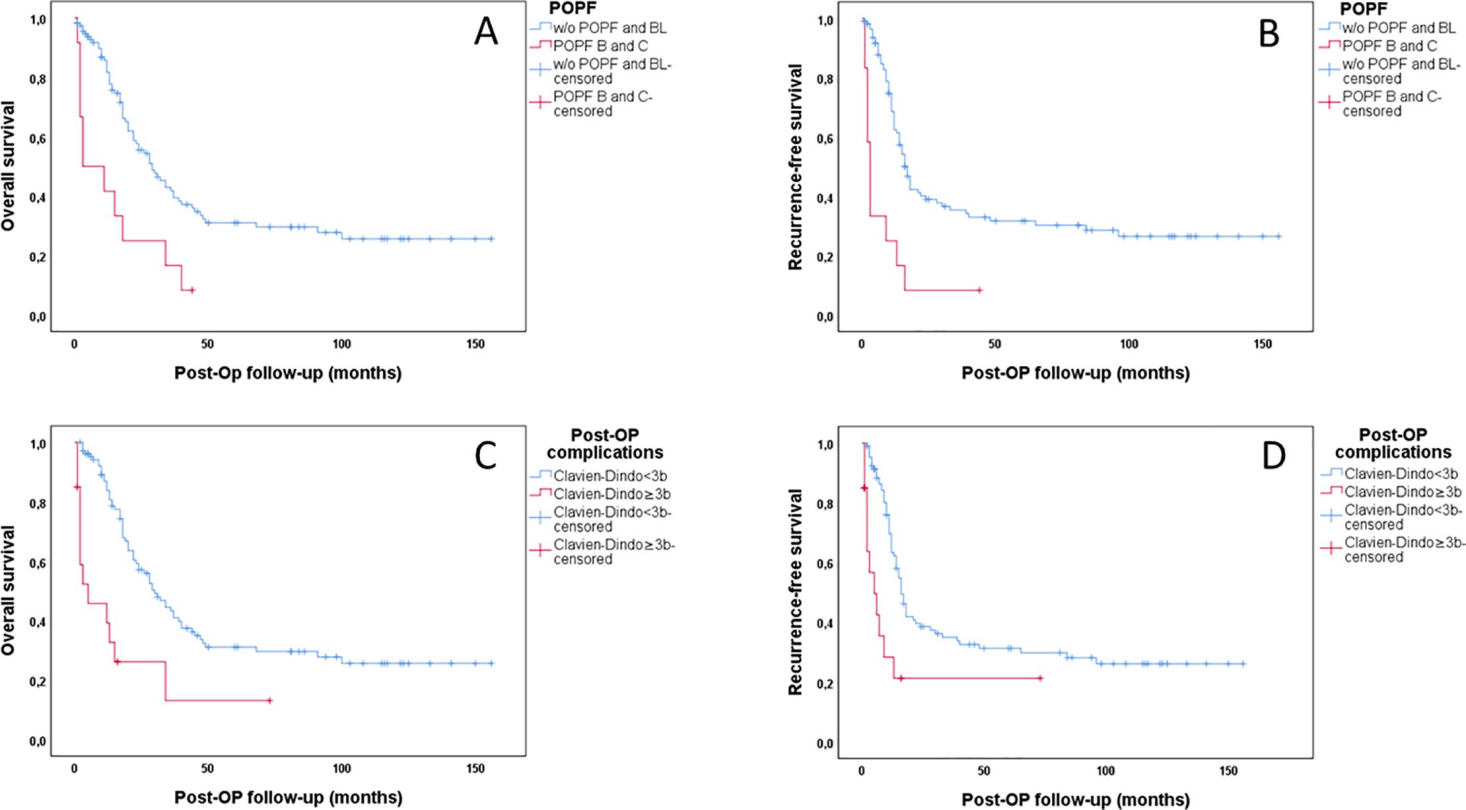
Kaplan-Meier analysis of overall and recurrence-free survival in PDAC patients. Prognostic impact of POPF (A, B) and postoperative major complications according to Clavien-Dindo (C, D) on overall survival (p = 0.002; p<0.001) and recurrence-free survival (p<0.001; p = 0.001) in curatively treated PDAC patients UICC Stages I to III.

Univariable Kaplan–Meier survival analysis showed a decreased overall survival (p = 0.002) and recurrence-free survival (p<0.001) in curatively treated PDAC patients UICC Stage I to III (n = 126) with POPF grade B or C versus PDAC patients without POPF or BL **(Fig 2A and 2B)**. Decreased overall survival (p<0.001) and recurrence free survival (p = 0.001) was associated with the occurrence of major postoperative complications of Clavien-Dindo ≥3b in curatively treated PDAC patients as well **(Fig 2C and 2D)**.

Univariable Cox regression analysis indicated improved overall and recurrence-free survival in PDAC patients with early UICC stage (p = 0.035; p = 0.048), low histological grading (p = 0.010; p = 0.002), low depth of invasion (p = 0.012; p = 0.013), and nodal invasion (p = 0.035; p = 0.048) as well as low NYHA score (p = 0.017; p = 0.049), elevated levels of pre-surgical tumor marker CA.19-9 (p = 0.012; p = 0.024), incidence of POPF grades B and C (p = 0.002; p<0.001), and postoperative major complications according to Clavian-Dindo ≥3b (p<0.001; p = 0.001) **(Tables 6 and 7)**. In the multivariable Cox analysis NYHA (p = 0.004), UICC stage (p = 0.012), tumor differentiation (p = 0.001), depth of invasion (p<0.001), nodal invasion (p = 0.044), and the incidence of postoperative major complications (p = 0.025) remained independent risk factors for overall survival. Prognostic markers of recurrence-free survival were UICC stage (p<0.001), tumor differentiation (p<0.001), depth of invasion (p = 0.001), nodal invasion (p = 0.001), postoperative major complications (p<0.001), and the incidence of POPF grade B and C (p = 0.006).

The incidence of clinically relevant POPF (HR no vs. yes [CI]: 0.100 [0.034–0.291]; p<0.001) and major postoperative complications (HR no vs yes [CI]: 0.148 [0.041–0.530]; p = 0.003) correlated with early tumor recurrence within 6 months.

**Table 6:** Uni- and multivariable Cox regression analysis of overall survival in PDAC patients.

Variable	Subset	Univariate analysis		Multivariate analysis	
		HR [95% CI]^1^^,^^2^	*p*	HR [95% CI]	*p*
Age (years)	<60/≥60	0.900 (0.572–1.415)	0.647		
Gender	Female/male	1.026 (0.656–1.606)	0.911		
Body mass index(kg/m^2^)	<18,>25/18-25	1.053 (0.676–1.642)	0.818		
ASA	1-2/3-4	0.718 (0.477–1.081)	0.113		
NYHA	1-2/3-4	0.453 (0.236–0.870)	**0.017**	0.348 (0.170–0.713)	**0.004**
Smoker	No/Yes	0.681(0.392–1.181)	0.171		
Alcohol	No/Yes	0.644 (0.203–2.043)	0.644		
Pre-surgical diabetes	No/Yes	0.991 (0.605–1.622)	0.971		
Pre-surgical pancreatitis	No/Yes	0.754 (0.387–1.469)	0.407		
Pre-surgical CA.19-9 (U/l)	<37/≥37	0.514 (0.306–0.866)	**0.012**	0.738 (0.478–1.141)	0.172
Pre-surgical CEA (ng/ml)	<4,6/≥4,6	0.833 (0.414–1.676)	0.609		
UICC stage	I-II/III-IV	0.598 (0.371–0.965)	**0.035**	0.107 (0.019–0.617)	**0.012**
Grading	G1-2/G3-4	0.559 (0.359–0.871)	**0.010**	0.497 (0.335–0.739)	**0.001**
T-stage	T1-2/T3-4	0.392 (0.188–0.817)	**0.012**	0.352 (0.202–0.613)	**<0.001**
Nodal invasion	N0/N1-2	0.598 (0.371–0.965)	**0.035**	5.899 (1.046–33.264)	**0.044**
Lymphatic invasion	L0/L1	0.709 (0.455–1.104)	0.128		
Vene invasion	V0/V1	1.184 (0.640–2.191)	0.590		
Resection margin	R0/R1	0.931 (0.550–1.575)	0.790		
Surgery	left/head	0.995 (0.581–1.704)	0.986		
Chemotherapy	No/Yes	0.962 (0.568–1.630)	0.886		
Pancreatic fistula (B/C)	No/Yes	0.362 (0.190–0.689)	**0.002**	0.779 (0.431–1.407)	0.408
Sirius red global score	A/B,C	1.077 (0.600–1.933)	0.805		
Clavian-Dindo	<3b/ ≥3b	0.283 (0.154–0.521)	**<0.001**	0.592 (0.374–0.937)	**0.025**

Bold values indicate significance (p≤ 0.05).

^1^ HR, hazard ratio

^2^ CI, confidence interval.

**Table 7:** Uni- and multivariable Cox regression analysis of recurrence-free survival in PDAC patients.

Variable	Subset	Univariate analysis		Multivariate analysis	
		HR [95% CI]^1^^,^^2^	*p*	HR [95% CI]	*p*
Age (years)	<60/≥60	1.092 (0.699–1.706)	0.698		
Gender	female/male	1.061 (0.681–1.655)	0.793		
Body mass index	<18, >25/18-25	1.020 (0.966–1.076)	0.478		
ASA	1-2/3-4	0.779 (0.520–1.166)	0.255		
NYHA	1-2/3-4	0.521 (0.272–0.997)	**0.049**	0.572 (0.279–1.172)	0.227
Smoker	No/Yes	0.713 (0.417–1.220)	0.217		
Alcohol	No/Yes	0.744 (0.283–2.117)	0.618		
Pre-surgical diabetes	No/Yes	1.072 (0.656–1.752)	0.782		
Pre-surgical pancreatitis	No/Yes	0.896 (0.461–1.740)	0.745		
Pre-surgical CA.19-9 (U/l)	<37/≥37	0.555 (0.333–0.924)	**0.024**	0.672 (0.441–1.024)	0.064
Pre-surgical CEA (ng/ml)	<4,6/≥4,6	1.057 (0.527–2.122)	0.875		
UICC stage	I-II/III-IV	0.614(0.379–0.995)	**0.048**	0.032 (0.006–0.189)	**<0.001**
Grading	G1-2/G3-4	0.503 (0.322–0.785)	**0.002**	0.484 (0.329–0.713)	**<0.001**
T-stage	T1-2/T3-4	0.395 (0.190–0.823)	**0.013**	0.417 (0.243–0.715)	**0.001**
Nodal invasion	N0/N1-2	0.614 (0.379–0.995)	**0.048**	19.375 (3.383–110.960)	**0.001**
Lymphatic invasion	L0/L1	0.794 (0.510–1.236)	0.307		
Vene invasion	V0/V1	1.316 (0.695–2.488)	0.399		
Resection margin	R0/R1	1.030 (0.594–1.785)	0.916		
Surgery	left/head	0.894 (0.529–1.511)	0.676		
Chemotherapy	No/Yes	1.031 (0.608–1.747)	0.910		
Pancreatic fistula (B/C)	No/Yes	0.263 (0.138–0.501)	**<0.001**	0.533 (0.339–0.837)	**0.006**
Sirius red global score	A/B,C	0.915 (0.515–1.628)	0.763		
Clavian-Dindo	<3b/≥3b	0.340 (0.182–0.634)	**0.001**	0.246 (0.138–0.504)	**<0.001**

Bold values indicate significance (p≤ 0.05).

^1^ HR, hazard ratio

^2^ CI, confidence interval.

Univariable Cox regression analysis revealed improved overall and recurrence-free survival in NET patients with adjuvant chemotherapy (each p = 0.003). Decreased recurrence-free survival was associated with major postoperative complications of Clavien-Dindo ≥3b (p = 0.022) in curatively treated NET patients. In patients with curatively treated papillary cacinoma, decreased overall and recurrence-free survival were associated with smoking (p = 0.002; p = 0.004), high histological grading (p = 0.036; p = 0.026), and no adjuvant chemotherapy (p = 0.031; p = 0.04). Multivariable Cox regression analyses revealed no significant risk factor for overall and recurrence-free survival in patients with NET or papillary carcinoma.

### Picrosirius red staining score

Histochemical staining by Picrosirius Red and its grading of fibrosis, inflammation, and steatosis were performed in pancreatic tissue sections of the resection margins of 178 consecutive study patients with PD since 2009. **Table 8** shows the distribution of the Picrosirius red global score and each of the three morphological features the score is based on in stained tissue samples by POPF grading. Low-grade fibrosis and high-grade inflammation or steatosis of the pancreatic remnant were associated with higher POPF grades (each p<0.01). A correlation between the Picrosirius red staining score and the incidence of severe postoperative complications and clinically relevant POPF was also found in the univariable logistic regression analyses (p≤0.001) (**Tables 3 and 4**). In multivariable logistic regression analyses, the Picrosirius red staining score correlated only with POPF (p = 0.049). Cox regression analysis for overall and recurrence-free survival revealed no prognostic impact of Picrosirius red global score in the study group (**Tables 6 and 7**).

**Table 8:** Assessment of picrosirius red staining score.

Grading	Total	w/o POPF n = 113 (63.5%)	Biochemical leak n = 29 (16.3%)	POPF B n = 18 (10.1%)	POPF C n = 18 (10.1%)	*p*-value
**Fibrosis**						
1 (>20%)	93 (52.2%)	81 (71.6%)	8 (27.5%)	1 (5.5%)	3 (16.6%)	**<0.001**
2 (11–20%)	39 (21.9%)	15 (13.2%)	10 (34.3%)	10 (55.5%)	4 (22.2%)	
3 (0–10%)	46 (25.8%)	17 (15%)	11 (37.9%)	7 (38.3%)	11 (61.1%)	
**Inflammation**						
1 (0–10%)	175 (98.3%)	113 (100%)	29 (100%)	16 (88.8%)	17 (94.4%)	**0.008**
2 (11–20%)	1 (0.5%)	0 (0%)	0 (0%)	1 (5.5%)	0 (0%)	
3 (>20%)	2 (1.1%)	0 (0%)	0 (0%)	1 (5.5%)	1 (5.5%)	
**Fat**						
1 (0–10%)	69 (38.7%)	54 (47.4%)	8 (27.5%)	1 (5.5%)	6 (33.3%)	**0.003**
2 (11–20%)	74 (41.5%)	44 (38.9%)	15 (51.7%)	8 (44.4%)	7 (38.8%)	
3 (>20%)	35 (19.6%)	15 (13.2%)	6 (20.6%)	9 (50%)	5 (27.7%)	
**Global Score**						
A (3–4)	85 (47.7%)	76 (67.2%)	7 (24.1%)	1 (5.5%)	1 (5.5%)	**<0.001**
B (5–6)	81 (45.5%)	36 (31.8%)	21 (72.4%)	11 (61.1%)	13 (72.2%)	
C (7–9)	12 (6.7%)	1 (0.8%)	1 (3.4%)	6 (33.3%)	4 (22.2%)	

Bold values indicate significance (p ≤ 0.05, Chi-Quadrat-test). POPF, postoperative pancreatic fistula; w/o, without.

## Discussion

In the last decades, advances in surgical techniques and in perioperative care, in addition to careful patient selection, have improved the outcomes in pancreatic surgery with considerable decrease of mortality rates below 5%. However, morbidity remains close to 50% even in high-volume centres, where clinically relevant POPF in 12 to 20% of patients continues to be the major cause of subsequent severe complications [2,3,34]. Numerous studies have identified a number of risk factors associated with an increased incidence of POPF including age, gender, BMI, pancreatic duct diameter, pancreas texture, operative time, and anastomosis technique [3,19,20,22,33]. Logistic regression analysis of clinicopathological parameters in our study cohort could confirm increased risk of major complications post pancreatic surgery in patients with advanced age, increased BMI and ASA, coexisting cardiopulmonary diseases, absence of preoperative chronic pancreatitis, intraoperative blood transfusion, prolonged operative and anaesthesia time, PD procedure, soft pancreatic tissue, and absence of internal or external pancreatic duct stenting. Furthermore, preoperative anemia and elevated serum levels of bilirubin and CRP were associated with postoperative complications. The multivariate correlation of postoperative major complications and elevated preoperative serum levels of CRP ≥8 mg/l reflects the systemic inflammatory response of the study patients with predominantly malignant and chronic inflammatory pancreatic diseases. However, the results suggest that even a subtle rise in preoperative CRP may stratify patients into a higher risk group for postoperative major complications. A recent study by Oehme et al. identified preoperative anemia as a risk factor for postoperative complications greater than grade 2 according to Clavien-Dindo and as an independent prognostic factor for shorter overall survival in patients undergoing surgical procedures for pancreatic malignancies [35]. A preoperative serum bilirubin level of >3 mg/dl was the most significant risk factor for clinically relevant POPF in a retrospective analysis by Rungsakulkij et al. [36] Univariable regression analysis revealed that the rise in the serum levels of amylase and lipase in the early postoperative period was associated with postoperative major complications suggesting manifestation of intraoperatively induced self-resolving pancreatitis [37]. In line with a multitude of reports, elevated amylase activity in the drainage fluid on POD 3 correlated with postoperative major complications, particularly with POPF [2,5,7,8,18,38,39]. The POPF definition by the ISGPS using amylase drain concentration is widely used. However, in accordance with our findings Tzedakis et al. could show that lipase is as effective as amylase drain concentration to define POPF [40]. Consequently, ICU and hospital stay were prolonged in patients with postoperative complications. In particular, the incidence of clinically relevant POPF correlated with a high score of histological Picrosirius red staining in multivariable analysis. The used Picrosirius red F3BA is a strong, linear anionic dye comprising six sulfonate groups that can associate along cationic collagen fibres. Picrosirius red staining developed by Junqueira et al. [31] provides a simple, specific, and sensitive method for localizing fibrillar collagen in tissue sections. In addition to the surgeon’s judgment about pancreatic texture by palpation, this histological tool allows a more objective assessment of the pancreatic remnant before reconstruction. There is broad agreement that fibrosis and low-grade fatty or inflammatory cell infiltration of the pancreatic parenchyma are POPF protective factors. It could be demonstrated that increased pancreatic fibrosis e.g. due to chronic pancreatitis not only enables a more secure anastomosis with solid fixing sutures but was also associated with decreased exocrine activity with reduced pancreatic juice output [32,33,41–43]. Therefore, intraoperative knowledge about the pancreatic texture in addition to the dignity of the resection margin of the pancreatic stump could help surgeons to adjust their surgical procedure. Besides the choice of the anastomotic technique, the use of intraoperative internal or external pancreatic duct stenting or even total pancreatectomy to prevent devastating POPF could be the consequence. We believe that further prospective studies evaluating the impact of intraoperative frozen section histology on anastomotic technique and outcome could help pancreatic surgeons prevent clinically relevant POPF.

In addition to patient characteristics and blood tests, the preoperative risk assessment of non-invasive cross-sectional imaging related to POPF has been the focus of several research groups [44,45]. A systematic review and meta-analysis of POPF prediction using preoperative computed tomography (CT) scan by Yue et al. revealed a significant increase in the incidence of clinically relevant POPF in patients with visceral obesity and sarcopenic obesity [46]. Furthermore, a narrow pancreatic duct assessed by preoperative CT images significantly related to POPF [47]. Multiparametric magnetic resonance imaging of the pancreas by Yoon et al. could enable accurate quantification of pancreatic fibrosis and steatosis, which have been shown to be associated with POPF [48]. The correlation of preoperative cross-sectional imaging with the surgeon’s judgment about pancreatic texture by palpation and intraoperative histological findings may allow a more objective assessment of the pancreatic remnant to prevent POPF and should be evaluated in future studies.

Although high grade POPF is universally regarded as a major source of early postoperative morbidity and mortality, its role in oncological outcome remains uncertain. Indeed, only a few studies have investigated the impact of clinically relevant POPF on PDAC specific survival and recurrence with contradictory results [6,49–51]. However, recent reports have demonstrated that early initiation of adjuvant chemotherapy was an important prognostic factor in patients with PDAC as severe POPF significantly prolonged initiation of adjuvant chemotherapy after primary surgery [52,53]. In curative resected PDAC patients of our study, Cox regression analyses indicated a survival benefit for low UICC and NYHA stages, low grading, low tumor invasion, absence of nodal invasion, low preoperative serum level of CA.19-9, and low incidence of postoperative major complications according to Clavien-Dindo. Advanced UICC stages and tumor differentiation, tumor invasion, nodal invasion, high preoperative serum level of CA.19-9, and the incidence of major complications, in particular clinically relevant POPF were identified as potentially favorable factors for tumor recurrence. Moreover, high grade POPF correlated with recurrence-free survival in multivariable analysis. Our results are in line with the negative influence of anastomotic leakage on survival outcome with high incidence of local recurrence in other gastrointestinal carcinoma entities [54]. It is supposed that anastomotic leakage leads to inflammation with the release of pro-inflammatory cytokines that alter host defense and promote growth of residual cancer cells [55]. In PDAC, Nagai et al. were able to demonstrate that clinically relevant POPF was an independent prognostic factor for peritoneal tumor recurrence [51]. Interestingly, even 28% of R0 curative resected patients with invasive PDAC revealed postoperatively higher cytology-positive rates in the drained fluid from the pancreatic bed with subsequent development of local recurrence [56].

Taken together, in addition to known risk factors the Picrosirius red staining comes along with a high diagnostic potential in the risk management of POPF that correlates with tumor recurrence in this study cohort. Further multicenter studies with a larger number of PDAC patients are required to reevaluate the value of intraoperatively extended histological diagnostic as well as the impact of high grade POPF as an independent survival predictor.

## Conclusion

This study indicates the high potential of pre-surgical risk stratification of known clinical risk factors and the intraoperative histopathological diagnostic of fibrosis, fatty and inflammatory cell infiltration in the resection margins of pancreatic stumps of curatively resected PDAC patients to prevent devastating POPF. Its prevention is urgently needed as clinically relevant POPF seems to be a prognostic factor of tumor recurrence in PDAC.
